# A Comparison of Direct and Two-Stage Transportation of Patients to Hospital in Poland

**DOI:** 10.3390/ijerph120504572

**Published:** 2015-04-24

**Authors:** Anna Rosiek, Aleksandra Rosiek-Kryszewska, Łukasz Leksowski, Krzysztof Leksowski

**Affiliations:** 1 Department of Public Health, Faculty of Health Sciences, Nicolaus Copernicus University in Toruń, Bydgoszcz 85-830, Poland; 2 Poland & Ross-Medica, Bydgoszcz 85-843, Poland; 3 Department of Inorganic and Analytical Chemistry, Faculty of Pharmacy, Nicolaus Copernicus University in Toruń, Bydgoszcz 85-089, Poland; E-Mail: ola.chemia@wp.pl; 4 Department of Rehabilitation, Faculty of Health Sciences, Nicolaus Copernicus University in Toruń, Bydgoszcz 85-094, Poland; E-Mail: leksowski.lukasz@wp.pl; 5 Department of Public Health, Faculty of Health Sciences, Nicolaus Copernicus University in Toruń, Bydgoszcz 85-830, Poland; E-Mail: leksowski@poczta.onet.pl

**Keywords:** transportation, two-stage transportation, hospital, patients, ECG transmission, telemedicine, public health

## Abstract

*Background*: The rapid international expansion of telemedicine reflects the growth of technological innovations. This technological advancement is transforming the way in which patients can receive health care. *Materials and Methods*: The study was conducted in Poland, at the Department of Cardiology of the Regional Hospital of Louis Rydygier in Torun. The researchers analyzed the delay in the treatment of patients with acute coronary syndrome. The study was conducted as a survey and examined 67 consecutively admitted patients treated invasively in a two-stage transport system. Data were analyzed statistically. *Results*: Two-stage transportation does not meet the timeframe guidelines for the treatment of patients with acute myocardial infarction. Intervals for the analyzed group of patients were statistically significant (*p* < 0.0001). *Conclusions*: Direct transportation of the patient to a reference center with interventional cardiology laboratory has a significant impact on reducing in-hospital delay in case of patients with acute coronary syndrome. *Perspectives*: This article presents the results of two-stage transportation of the patient with acute coronary syndrome. This measure could help clinicians who seek to assess time needed for intervention. It also shows how time from the beginning of pain in chest is important and may contribute to patient disability, death or well-being.

## 1. Introduction

Technological advances have begun to revolutionize the ways in which hospital facilities are operated. According to Gaseau [1], medical institutions and other organization are beginning to use technology as a manpower multiplier, and technology provides an opportunity to enhance the safety of employees and the safety of patients [1,2]. Technological advances that are widely used and have been found to be effective are surveillance and security technologies, tracking and detection devices, the use of automated management technologies, and communication technologies, which include teleconferencing and fast methods of professional communication in medicine. These telecommunication technologies help to bridge distance and support heath care delivery.

The current European and Polish market for telemedicine is growing rapidly. The rapid international expansion of telemedicine reflects the growth of technological innovations [3,4,5]. High-capacity digital networks, powerful and affordable computer hardware and software, high-resolution digital images, and the Internet have had a great impact on the process of health care delivery. These technological advances are transforming the way in which patients receive health care and has significant implications with regard to financial costs, ensuring the security of patients and medical staff comfort of work. 

Telemedicine is a general term that refers to a wide range of technologies and applications in medicine and especially in medical treatment processes. In a broad sense it is defined as the use of medical information exchanged from one site to another via electronic communications for the health and education of the patient or health care provider with the purpose of improving patient care. It includes services using two-way video, email, smart phones, wireless devices and other forms of telecommunications technology [6]. An example of the use of telemedicine is the use of information technology and telecommunications to bridge geographical distances and improve health care delivery. 

Telemedicine refers to the actual delivery of remote clinical services using technology. The American Telemedicine Association (ATA) describes telemedicine not as a separate medical specialty, but rather products and services which are part of a larger investment by health care institutions both in information technology and the delivery of clinical care [6].

**Figure 1:**
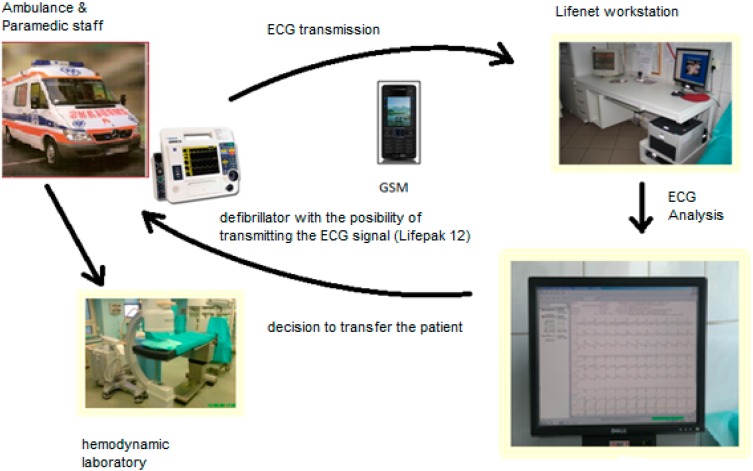
Teletransmission system in Poland. The ambulance team performs ECG using a *Lifepack 12* defibrillator. Then, using the mobile station they transmit a record via Lifenet to a hospital with interventional cardiology. The doctor on duty at the cardiology ward, after analyzing the transmitted data (record) and after a telephone conversation with the paramedic ambulance staff, makes a decision on the transportation of patient to the hospital with interventional cardiology. Source: the authors’ own study.

With the technological development observed in medicine more and more information can be transmitted electronically in a shorter time. This form of information and data transfer has contributed to the development of various medical procedures such as telerehabilitation, ECG teletransmission, telemonitoring, radiological teleconsultation, teleconsultation doctor-doctor, doctor-patient teleconsultation, education, *etc.* All these procedures are included in the wider term telemedicine. Telemedicine holds great promise in facilitating emergency medical practice. It is particularly well suited to medical emergencies where treatment delays can adversely affect clinical outcome. A typical scenario is ST elevated myocardial infarction (STEMI) where recognition of ECG changes by paramedics could facilitate early intervention and improve clinical outcome [7]. Paramedics in ambulances can use telemedicine links with specialists to facilitate pre-hospital diagnosis and reduce delays in myocardial infarction, stroke and trauma. The use of telemedicine in both in myocardial infarction [7,8,9] and stroke management is feasible [10,11] but dependent on the technical performance of the telemedical equipment and broadband infrastructure [12,13]. The Figure 1 below shows a telemedical infrastructure used in Poland.

**Figure 2:**
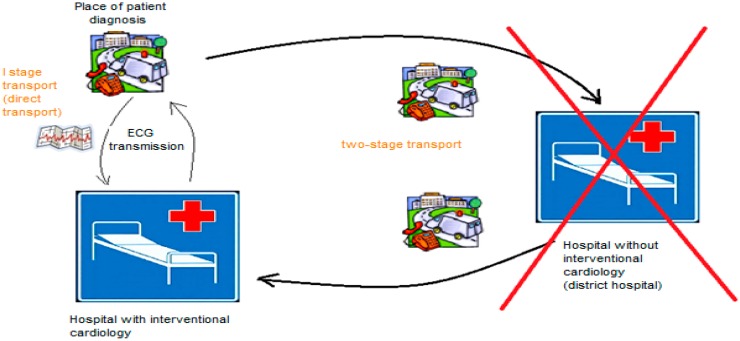
Scheme of a two-stage transport and one-stage transport of patients to a hospital model from Poland. Source: authors’ own study.

The hub and spoke model used in EMS provides an ideal opportunity for supervision by a centrally located expert (hub). Analysis of clinical outcomes of patients managed using this model (Lifenet system) suggests that although there is increased consultation, the quality of care is higher and saves time necessary to start medical intervention, and reduces transport costs [14]. With the development of the Lifenet network in Poland, it is possible to transfer the ECG performed on a patient with chest pain remotely using a Lifepak12/Lifepak15 defibrillator. On this basis, the decision is made to transport the patient directly (I stage transport) to the reference center eliminating the two-stage transport to the hospital. Patients are transferred directly to the reference laboratory after pre-hospital diagnosis of ST elevation myocardial infarction (STEMI) in a telemedicine equipped ambulance to singular tense treatment delay [9,15] and patients’ mortality caused by myocardial infraction [16] (Figure 2). 

Telemedicine technologies have great potential to provide expertise from the hospital to the personnel at the emergency site and patient-relevant information to the receiving hospital [17,18]. This potential has been recognized by the European Stroke Initiative (EUSI) [19] that not only recommended the activation of a “code of impact” as a special infrastructure with direct connection to the stroke unit neurologist and direct transport to the resort for treating strokes, but also the use of telemedicine in rural and remote areas in order to improve access for treatment [13,20,21,22].

Many studies have shown the reliability of telemedicine-based interaction between rural hospitals and stroke centres [18,23,24]. Thus, nonspecialised hospitals have the option to obtain guidance from high-level stroke centers via systems with the ability to provide two-way real-time audiovisual conferencing and sharing of images [25,26]. The results of several trials have shown not only the reliability and safety of remote clinical decision making [27,28,29,30], but also its positive effects on thrombolysis rates and clinical outcome [31], similar to cardiology patients with myocardial infarction.

There are significant positive outcomes of telemedically managed stroke [10,11,12,32]. The development of telemedicine programs for the treatment of stroke requires capital investment in infrastructure, which includes computer hardware and related software, audiovisual equipment, and high-speed bandwidth that can support high quality video. Available funding for this technology is limited, so Poland is positioned at a very early stage of telemedicine implementation until more evidence of region- and program-specific clinical and cost effectiveness is accumulated. Undoubtedly, direct transfer of the patients by ambulance to the stroke unit is the best option in the case of stroke [20,22] and STEMI [21].

Telemedicine includes the use of advanced telecommunications technologies to exchange health information and provides health care services to cardiology patients across time, social and cultural barriers and geographic locations [33]. Telemedicine was adopted quickly in cardiology and was used frequently also in several medical specialty areas (emergency, neurology, oncology). Telemedicine improves such quality care indicators as: time between specialty referral and access to a higher level of care [34]. The capabilities of telemedicine in cardiology settings offer the opportunity of expanding access to health care providers. Information about persons who need treatment or diagnostic services can be transmitted rapidly to physicians or other health care providers located in other parts of the country, as well as provide access to medical specialists. For example, through the use of data (ECG) patients with suspected acute coronary syndrome can be diagnosed by a specialist at a distance, which in turn reduces the time of referring the patient to the cardiac catheterization laboratory by about 50–80 min [4,35,36,37]. The introduction of asset such as: pre-hospital ECG data transmission facilitates quick identification of patients with acute myocardial infarction and referring the patient to the proper hospital, as well as of reducing the time of “door-to-balloon” path.

## 2. Materials and Methods

The study was conducted in Poland in the Kujavian-Pomeranian region, at the Department of Cardiology of the Regional Hospital of Louis Rydygier in Torun. The researchers analyzed the delay in the treatment of patients with acute coronary syndrome. The study examined 67 consecutively admitted patients treated invasively in two-stage transport system. The analysis included only patients referred urgently (on admission) and classified for emergency invasive diagnostics mode during the analysed period—from March to May 2011. Patient demographics are shown in Table 1. The patients came Golub Dobrzyń, Brodnica, Toruń (40 patients, Toruń area), Wąbrzeźno, Chełmża, Grudziądz, Chełmno (25 patients, Grudziądz area), and Lipno, Włocławek (2 patients, Włocławek area). By gender 67.2% were male and 32.8% female. Ages ranged from 46 to 69 (average 57.5). 

**Table 1:** Patients’ Demographic Data. Sex: In the STEMI and NSTEMI groups the majority of patients were men. In the UA group the majority of patients were women. The gender difference between the groups is statistically significant. Education: Analysis of patients according to the education level, showed that in all the groups patients with elementary level and basic technical education prevailed. The difference in education level between the groups of patients was not statistically significant.

Two-Stage Transport to a Referral Hospital									
**Sex**	**STEMI**		**NSTEMI**		**UA**		**Sum**		***p***
	**No.**	**%**	**No.**	**%**	**No.**	**%**	**No.**	**%**	
Female	10	14.9	10	14.9	2	3.0	22	32.8	*p* < 0.05
Male	21	31.4	23	34.3	1	1.5	45	67.2	
Sum	31	46.3	33	49.2	3	4.5	67	100	
**Education Level**	**STEMI**		**NSTEMI**		**UA**		**Sum**		***p***
	**No.**	**%**	**No.**	**%**	**No.**	**%**	**No.**	**%**	
Elementary Level	8	12.0	11	16.4	1	1.5	20	29.9	*p* > 0.05
Basic Technical	16	23.9	13	19.4	2	3.0	31	46.3	
High School	6	8.9	2	3.0	0	0	8	11.9	
University Level	1	1.5	7	10.4	0	0	8	11.9	
Sum	31	46.3	33	49.2	3	4.5	67	100	

The study shows the analysis of time delays both dependent (the interval from symptom onset to first call for medical help is the dominant part of the delay) and independent (delays in treatment at the level of ambulance and emergency doctors—because of the lack of priority for the direct transport, inefficient in-hospital care). The study was designed to determine the ability of a two stage (transport with incorrect diagnosis requiring second transport to an appropriate hospital) *versus* a one stage (transport to an appropriate hospital with correct diagnosis by using telemedicine) system to comply with the 90 min standard. Data was statistically analyzed. Wilcoxon tests in the analysis groups were confirmed and reached statistical significance (*p* < 0.0001). It was also noted in the group that there was a low percentage of cases in which acute coronary syndrome was excluded (0.71%) by no transmission. Wilcoxon tests also confirmed the statistical significance *p* < 0.0001 of two aspects of two-stage transportation of patients to hospital:

- the time of admission to the hospital (time from the beginning of transportation from district hospital to reference center ) and the start of coronary angiography—average time was 140 min and,
- the time of admission to the hospital and the start of coronary angioplasty—average time was 95 min.

In the analyzed group, where ECG signals were transmitted, intervals were statistically significant (*p* <0.0001). The Institutional BioMedical Ethics Committee approved this study. Written informed consent for participation in the study was obtained from participants of the study and for publication of this report. 

## 3. Results

**Figure 3:**
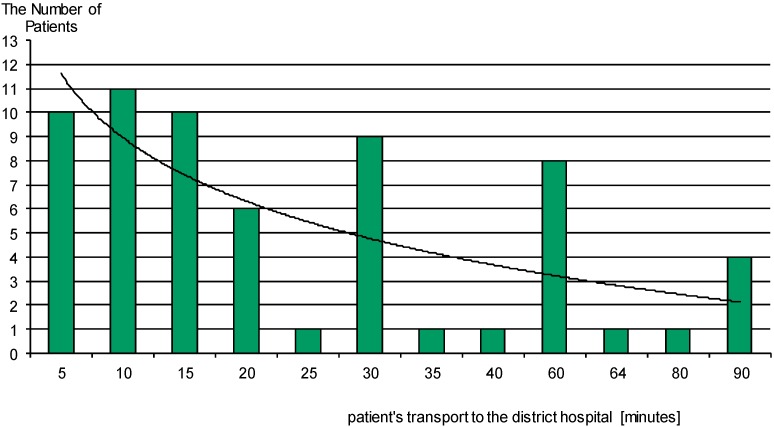
Transportation time of patients with acute coronary syndrome to the district hospital. Patient transport time to the district hospital (stage I transport) ranged from 5 to 90 min. Average patient transport time in the first stage of transport to the hospital is about 28 min.

**Figure 4:**
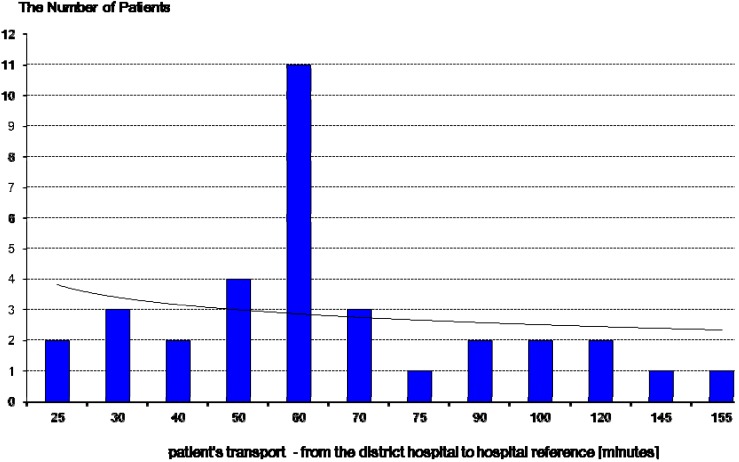
Transportation time of patients with acute coronary syndrome from the district hospital to the referral hospital (stage II transport). Patients’ transportation times from the district hospital to the referral hospital (two—stage transport) ranged from 25 min to 155 min. Average time to the two-stage reference center was about 67 min.

**Figure 5:**
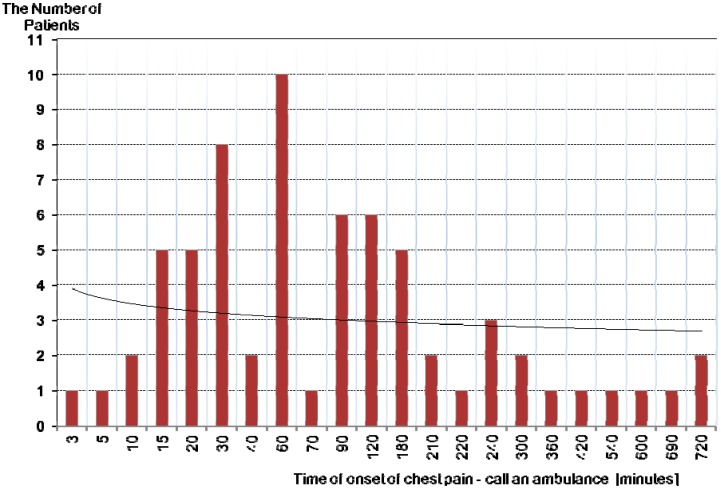
The time dependent on the patient—from the beginning of the chest pain until medical help. Time of providing medical help (depending on the patient) ranges from 3 to 720 min. The average time was 140 min.

The results of this study showed time delays, both dependent and independent of the patient. Delays independent of the patient, resulting from the use of two-stage transport to a referral hospital, are shown in Figure 3 and Figure 4 and Table 2 below. Time delays dependent on the patient is shown in Figure 5.

**Table 2:** Comparison of time delay between one and two-stages of hospital transport.

Two-Stage Transport to a Referral Hospital	Timedelays Sume (Dependent and Independent)	Group	*p*
		*N* = 67	
STEMI	95 min	31	<0.0001
NSTEMI	N/A	33	0.2692
UA	140 min	3	<0.0001
Diagnosis with using technology (One-stage transport)	28 min	67	N/A

The vulnerable point in the chain of events leading to prompt and effective treatment is patient delay in seeking care. This is a worldwide tendency which is also evident in Poland [9,21,22,35]. Factors such as old age, female sex, low education level, low socioeconomic status, and place of living (e.g., countryside) are associated with increased delays in seeking treatment [5,14,21,38,39]. In the studies group ECG was performed but not transmitted to the reference center and transport took place in two stages. Intervals for the analyzed group of patients were statistically significant (*p* < 0.0001). In 53% of the analyzed group, it was found that the time that elapsed since admission to the start of coronary angioplasty in referral center was more than 90 min. In addition, it should be noted that the weak point in the process of treating a patient with acute coronary syndrome, proved to be patients who decided to call an ambulance 140 min (average time) after the onset of pain. Public education through mass media could be increase public awareness of acute coronary syndrome and could reduce delay in presenting to the ECG data to the reference center. 

## 4. Discussion 

Two-stage transportation does not meet the timeframe guidelines for the treatment of patients with acute myocardial infarction (less than 90 min from ambulance arrival to start of treatment). Other researchers have also shown that the time lost in transportation and two step-hospital delay time is too long to fit in the given time frame to begin treatment [5,40]. This time is important and recommended in accordance with the guidelines of cardiology of the European Society of Cardiology and the Polish Cardiac Society of 2012. Patients with a diagnosis of acute coronary syndrome and ST-segment elevation should, in accordance to current standards, receive reperfusion therapy within 90 min since the first medical contact. In-hospital delay (in the hospital with interventional laboratory) should not exceed 60 min. 

American Heart Association Guidelines for Cardiopulmonary Resuscitation and Emergency Cardiovascular Care from 2010 also suggest that post-cardiac-arrest hospital care has significant potential to reduce early mortality caused by hemodynamic instability and later morbidity and mortality from multi-organ failure and brain injury [37,41,42]. Therapeutic hypothermia and treatment of the underlying cause of cardiac arrest have impact on survival outcome [41,43]. Hemodynamic optimization and multidisciplinary early goal-directed therapy have been introduced as part of a bundle of care to improve patient outcome and to ensure better organ perfusion and oxygenation of organs [41,44,45,46] and give the positive treatment results. The experiences from Poland (in Cracow’s hospital relating to the rescue of a boy after stopping the circulation) [44,47], in particular show how important is this activity. Thereby topics analyzed and discussed taken by author’s in paper are current.

The most critical time of a STEMI is the very early phase, during which the patient is often in severe pain and liable to cardiac arrest. Furthermore, the earlier some treatments, notably reperfusion therapy, are given, the greater the beneficial effect (“time is muscle”). Yet, it is often an hour or more after the onset of symptoms before medical aid is requested. Older patients, female, diabetic, and congestive heart failure patients are more likely to delay seeking care [48,49]. 

Because of that, the ambulance service has a critical role in the management of a STEMI [50], and should be considered not only a mode of transport but a place for initial diagnosis, triage, and treatment [4]. Ambulances should be able to reach the great majority of chest pain patients within 15 min of the call [51]. The quality of the care given depends on the training of the staff in an ambulance. At the most basic level, all ambulance personnel should be trained to recognize the symptoms of a STEMI, administer oxygen, relieve pain, and provide basic life support and direct the transport of a patient to the reference center as fast as possible.

As indicated above, the implementation of a network of hospitals connected by an efficient ambulance service and using a common protocol to patient’s diagnosis is key for an optimal management of patients with STEMI [49].

With such a network in place, target delay times should be: <10 min for ECG transmission; ≤5 min for tele-consultation; ≤90 min since ambulance arrival to first balloon inflation. A greater delay in the treatment is associated with an increased mortality ratio within six months following the treatment among patients who received fibrinolytics and among those treated by primary PCI. Quality of care, appropriateness of reperfusion therapy, delay times, and patient outcome should be measured and compared at regular intervals and appropriate measures for improvement should be taken. 

The passage of time has an impact on prognosis. This fact is recognized by European (ESC) and American Societies of Cardiology (ACCF/AHA), which recommended that an ambulance rushing to a patient with a heart attack is equipped with a 12 lead ECG [5,40]. Attention to minimizing delays in the treatment of myocardial infarction with ST-segment elevation (STEMI) is crucial for two reasons:

- The most critical moment occurs at an early stage when the patient is in pain and is at risk of sudden cardiac arrest.
- Early reperfusion minimizes the effects of the so-called myocardial infarction (infarction scar).

Minimalization of the delay increases the quality of care. Bashshur [52] suggests that the quality of care provided by telemedicine in treatment of cardiology patients can be evaluated according to biomedical indicators such as clinical efficiency, effectiveness of therapy and patient’s safety [53]. Positive impact of the use of telemedicine in cardiology demonstrates how technology (teleheath) influences patient management therapy and how it can contribute to improving patients’ health and well-being after hospitalization process. 

Generally, outcomes of telemedicine in cardiology practices seem to show a positive trend [3], but medical benefits need to be monitored [54]. Indeed, some favorable aspects of telemedicine seem hard to refute, such as offering access to healthcare in rural areas or garnering ancillary savings by avoiding added travel time and cost in treatment of acute coronary syndrome [55]. 

Darkins and Cary (2002) identified important clinical aims of telemedicine [56] in treatment process of cardiology patients. They show, among other things, that the rapid and safe transfer of the patient to the hospital as a result of the use of advanced telecommunication technologies and exchanging health information, provides better quality of health care services. Thus, indicate that shorter in-hospital delays affect patient’s safety and quality of care. On the other hands, a well-functioning national and regional system of care based on pre-hospital diagnosis, triage and fast transport to the most appropriate facility is key to the success of the treatment, and significantly improves the outcome for patients with acute coronary syndrome [36,57]. 

Continuous advancements in telecommunication and telemedicine, the use of electronic signals to transfer medical data from one site to another via the internet, intranet, satellites, or videoconferencing telephone equipment significantly, help to improve access to health care [2,58]. 

We recommend further observation and study on the development of this area that includes wide range of services delivered, managed, and coordinated by all health-related disciplines via electronic information and telecommunications technologies [59]. 

## 5. Conclusions

Telemedicine has the potential to provide access to high quality medical care for isolated populations, empower patients to play active role in their disease management and could in some cases decrease the cost of care. Cyberspace has become an important territory for patients, practitioners, medical institutions, and policymakers. 

The use of new technologies in telemedicine allows for early transmission of the ECG form a patient with chest pain at the place of assistance. This fact contributes significantly to reducing the time delay of in-hospital coronary angiography and angioplasty. Direct transport of a patient to a reference center with interventional cardiology laboratory has a significant impact on reducing in-hospital delay for patients with acute coronary syndrome. 
